# Systemic *Exophiala equina* infection in an Eastern box turtle (*Terrapene carolina carolina*): a case report and literature review

**DOI:** 10.3389/fvets.2023.1158393

**Published:** 2023-05-12

**Authors:** Daniel Felipe Barrantes Murillo, Stephanie Anderson, Christian Capobianco, Gregory A. Lewbart, Nathan P. Wiederhold, Connie F. Cañete-Gibas, Tatiane Terumi Negrão Watanabe

**Affiliations:** ^1^Department of Pathobiology, College of Veterinary Medicine, Auburn University, Auburn, AL, United States; ^2^Department of Population Health and Pathobiology, College of Veterinary Medicine, North Carolina State University, Raleigh, NC, United States; ^3^Department of Clinical Science, College of Veterinary Medicine, North Carolina State University, Raleigh, NC, United States; ^4^Fungus Testing Laboratory, Department of Pathology and Laboratory Medicine, University of Texas Health Science Center at San Antonio, San Antonio, TX, United States; ^5^Antech Diagnostics, Los Angeles, CA, United States

**Keywords:** *Exophiala equina*, phaeohyphomycosis, systemic mycosis, eastern box turtle, reptile

## Abstract

Phaeohyphomycosis is an infection caused by melanized fungi. This disease has been reported in several animal species including invertebrates, cold-blooded vertebrates, mammals, and humans. Melanized fungi have similar phenotypical features and confirmation requires culture and molecular diagnostics. To exemplify this we present a case of a 333 g adult of unknown age, free-ranging, male Eastern box turtle (*Terrapene carolina carolina*) that was referred to the Turtle Rescue Team at North Carolina State University for evaluation of multilobulated masses occupying the entire left orbit and at the right forelimb on the plantarolateral aspect of the foot. A fine needle aspirate cytologic examination of the mass on the right forelimb revealed large numbers of inflammatory cells and fungal organisms. Histopathology of the skin biopsies from the right forefoot was consistent with phaeohyphomycosis. A course of antifungal medication was started (Fluconazole 21 mg/kg loading dose IV then 5 mg/kg PO SID q 30 days). Due to concern for the patient's quality of life and the lack of a curative treatment plan, humane euthanasia was elected. Gross and histological postmortem examination confirmed the presence of multiple coelomic masses similar in appearance to those observed in the left orbit and right forefoot indicating disseminated phaeohyphomycosis. A swab of the periocular mass was submitted for fungal culture and phenotypic identification. The isolate was later identified as *Exophiala equina* through a combination of phenotypic characterization and sequencing of the ITS region of the nuclear rDNA. *Exophiala* is a genus in the family Herpotrichiellaceae, order Chaetothyriales and is considered an opportunistic “black yeast” causing infection in aquatic invertebrates, fish, amphibians, reptiles, and mammals including humans. *Exophiala equina* is infrequently reported in animals, with only three cases in the literature including the herein report.

## 1. Introduction

*Exophiala* is a genus in the family Herpotrichiellaceae, order Chaetothyriales, that produces opportunistic “black yeast” by budding an annellidic conidiogenesis (1). *Exophiala* species are associated with opportunistic infection in animals and humans. The most relevant species usually reported as pathogens in humans are *Exophiala dermatitidis, E. spinifera*, and *E. asiatica* (1). *Exophiala* spp. infections also have been reported in crustaceans (*Ucides cordatus*), captive and farmed fish (*Salmo salar, Mustelus canis*, among others), amphibians (*Hyla caerulae, H. sptentrionali, Phyllobatest rinitatis*, among others), aquarium animals, and other cold-blooded vertebrates (2). *Exophiala* spp. infection is also reported in humans and mammals including dogs, cats, and a horse (2). Table 1 summarizes the selected cases of *Exophiala* spp. infection in vertebrate animals. Cold-blooded vertebrates usually develop a systemic infection, unlike mammals where localized skin or subcutaneous infections are reported. *E. equina* was first described as the causative pathogenic agent in a lower limb infection of a horse (3) and to our knowledge, it has not been described in mammals since then. There is a report of systemic infection caused by strain CBS 116009 of *E. equina* causing disseminated infection in a Galapagos giant tortoise (*Geochelone nigra*) (4). Other reported species of *Exophiala* causing disease in turtles are Aldabra tortoise (*Geochelone gigantea*) and Eastern box turtle (*Terrapene carolina carolina*) infected by *Exophiala oligospermia* and *Exophiala jeanselmei* respectively (5, 6). There is a case of oral granuloma and disseminated granulomas caused by *Exophiala* spp in a Radiated tortoise (*Astrochelys radiata)* (7). To our knowledge, this is the first report of *Exophiala equina* infection in an Eastern box turtle (*Terrapene carolina carolina*) and the third reported infection in animals.

**Table 1:** Selected cases of *Exophiala* infection in vertebrate animals.

**Host species**		**Fungal species**	**Type of infection**	**Reference**
Fish	Catfish (*Ictalurus punctatus*)	*Exophiala pisciphulus*	Systemic infection	(8)
	Atlantic salmon (*Salmo salar)*	*Exophiala salmonis*	Systemic infection	(9)
	Striped jack (*Pseudocaranx dentex*)	*Exophiala xenobiotica*	Systemic infection (gill, kidney, heart)	(10)
	Atlantic cod (*Gadus morhua*)	*Exophiala angulospora*	Systemic infection	(11)
	Atlantic halibut (*Hippoglossus hippoglossus*)	*Exophiala angulospora*	Systemic infection	(12)
	Bloch (*Epinephelus lanceolatus*)	*Exophiala xenobiotica*	Swim bladder infection	(13)
	Pretty tetra (*Hemigrammus pulcher*)	*Exophiala pisciphila*	Swim bladder infection	(14)
	Cardinal tetra (*Paracheirodon axelrodi*)	*Exophiala pisciphila*	Systemic infection (intestine, kidney, spleen)	(15)
	Lumpfish (*Cyclopterus lumpus*)	*Exophiala angulospora*	Systemic infection	(16)
	Weedy seadragon (*Phyllopteryx taeniolatus*)	*Exophiala angulospora* *Exophiala* sp. *nov*	Systemic infection	(17)
	Leafy seadragon (*Phycodurus eques*)	*Exophiala* sp. *nov*	Systemic infection	(17)
	Dogfish (*Mustelus canis*)	*Exophiala pisciphila*	Systemic infection (brain and skin)	(18)
	Zebra shark (*Stegostoma fasciatum*)	*Exophiala pisciphila*	Systemic infection	(19)
	Swell shark (*Cephaloscyllium ventriosum*)	*Exophiala* spp.	Skull and cervical vertebrae localized infection	(20)
Amphibians	European blind cave salamander (*Proteus anguinus*)	*Exophiala salmonis*	Systemic infection	(21)
	Common toad (*Bufo bufo*)	*Exophiala* sp.	Systemic infection (hepatitis, coelomitis)	(22)
	Estern hellbender (*Cryptobranchus alleganiensis alleganiensis)*	*Exophiala* novel species related to *Expohiala cancerae*	Systemic infection (multicentric granulomatous inflammation)	(23)
Reptiles	Radiated tortoise (*Astrochelys radiata*)	*Exophiala* spp.	Systemic infection: oral mass and disseminated granulomas	(7)
	Galapagos tortoise (*Geochelone nigra*)	*Exophiala equina*	Systemic infection (hematogenous spread)	(4)
	Eastern box turtle (*Terrapene carolina carolina*)	*Exophiala jeanselmei*	Localized subcutaneous infection	(5)
	Aldabra tortoise (*Geochelone gigantea*)	*Exophiala oligospermia*	Localized carapace infection	(6)
Birds	Wild turkey (*Meleagrides gallopavo*)	*Exophiala* spp.	Feather damage	(24)
Mammals	Cat (*Felis catus*)	*Exophiala jeanselmei*	Skin infections Subcutaneous infection Systemic infection	(25–28)
	Cat (*Felis catus*)	*Exophiala spinifera*	Skin infection Nasal infection	(29)
	Cat (*Felis catus*)	*Exophiala attenuata*	Skin infection	(30)
	Dog (*Canis lupus familiaris*)	*Exophiala dermatitidis*	Skin infection Systemic infection (peritoneum, right scapula)	(31, 32)
	Pig (*Sus scrofa domestica*)	*Exophiala jeanselmei*	Abortion	(33)
	Horse (*Equus caballus*)	*Exophiala equina*	Skin and subcutaneous infection	(3)

## 2. Case presentation

**Figure 1:**
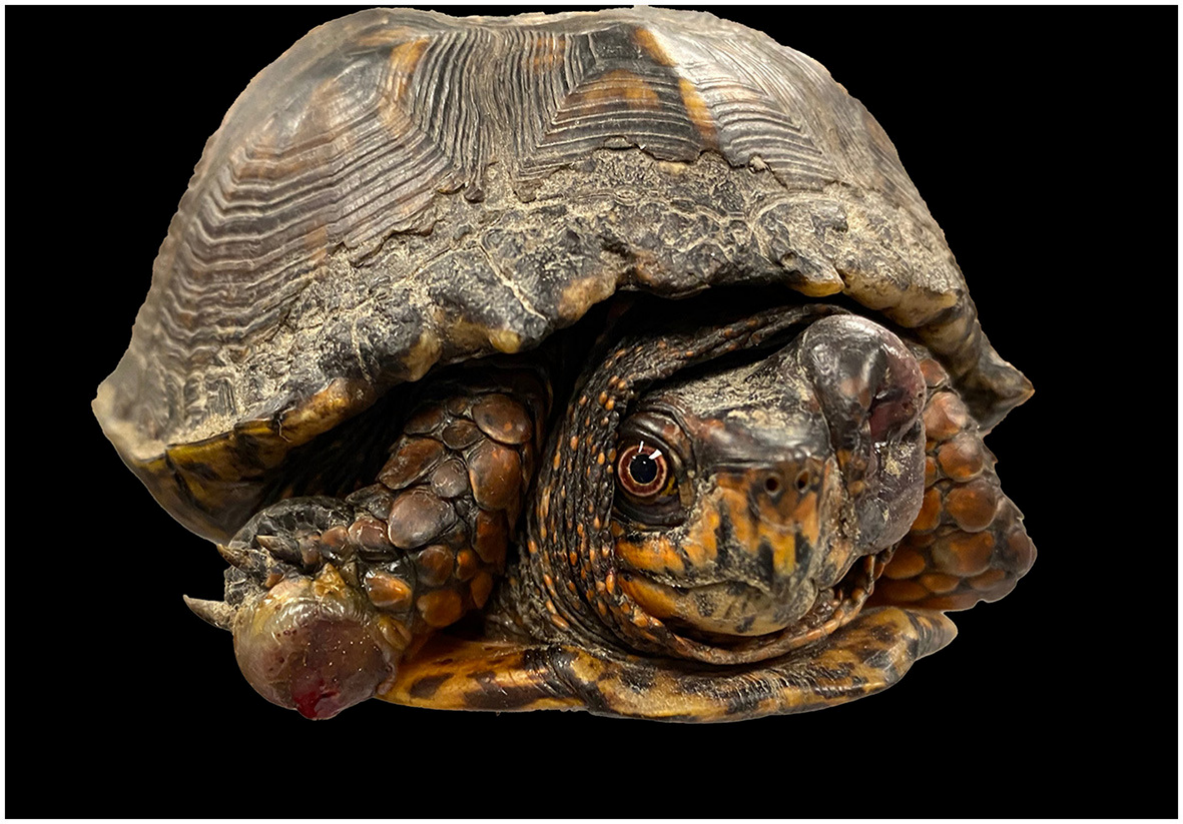
An adult of unknown age, intact male Eastern box turtle (*Terrapene carolina carolina*) with a mass was observed occupying the entire orbit of the left eye and right forelimb on the plantar and lateral aspect of the foot.

A 333 g, adult of unknown age, free-ranging, male Eastern box turtle (*Terrapene carolina carolina*) was referred to the Turtle Rescue Team (TRT) at the College of Veterinary Medicine, North Carolina State University for evaluation of multiple proliferative growths on the right foreleg and the left orbit. On physical examination, the turtle was uncomfortable on palpation of the masses. One mass was occupying the entire left orbit and along the right forelimb on the plantar and lateral aspect of the foot (Figure 1). The clinical management timeline is summarized in Table 2. There was no purulent material on aspiration of the forelimb lesion, however reptile's abscesses have caseous debris, thus the lack of purulent exudate did not rule out an abscess. Given the history of a free-ranging animal, uncomforted upon palpation and a possible caseous abscesses an empiric treatment with a non-steroidal anti-inflammatory drug (ketoprofen 2 mg/kg IM SID for 3 days) and antibiotic medication (ceftazidime, 20 mg/kg, IM, q96hrs, 4 doses) was prescribed at the time of initial evaluation to provide analgesia and cover a potential bacterial infection. The treatment was instituted until the patient was more comfortable to perform cytology and biopsy of the lesions. Five days after the initial presentation, a full physical examination was performed under sedation with alfaxalone (10 mg/kg IV) and morphine (1.5 mg/kg IM). A fine needle aspirate of the mass from the right forelimb was taken. Cytologic examination revealed abundant numbers of macrophages and heterophils, and multinucleated giant cells with numerous intracellular and extracellular, 5–15 μm diameter, round to ovoid elongated, occasionally forming chains, fungal organisms with a refractile wall. Two punch samples of the mass along the right forelimb were taken for histological evaluation. Histopathology of skin biopsies identified heterophilic granulomatous dermatitis, and panniculitis with intralesional pigmented fungal hyphae (consistent with phaeohyphomycosis). On day 13, a course of antifungal medication was started following the results of the cytology and the histopathology reports (fluconazole 21 mg/kg loading dose IV then 5 mg/kg PO SID q 30 days). Fluconazole was chosen due to the previous pharmacokinetic studies supporting its use in marine turtles for treating fungal infections and for being readily available in an oral formulation for use in TRT (34, 35). On day four after starting the antifungal treatment, the turtle was reluctant to medicate PO and the medication was injected into a mealworm and fed to the patient. At the end of the antifungal treatment, the turtle was still eating, drinking, defecating, and urinating. The bloodwork yielded a PCV of 17% (reference value: 8–34%) and total solids were 8.0 g/dL (reference interval: 3–10.6 g/dL) (36). However, there was no resolution of the lesions. The masses were larger, and the lesion on the left eye spread caudally. A CT scan was performed, and the fungal infection had spread to the lungs and other organs. Due to poor prognosis, humane euthanasia was elected 6 weeks after the initial evaluation. The animal was sedated by applying an alfaxalone IV (10 mg/kg) at the subcarapacial plexus. After sedation euthanasia was applied by injecting euthanasia solution of sodium pentobarbital IV (100 mg/kg) at the post-occipital sinus. Death was confirmed by lack of deep pain response, lack of corneal reflex, rigor mortis, and absence of Doppler heart rate.

**Table 2:** Clinical management timeline of a free-ranging, male Eastern box turtle (*Terrapene carolina carolina*) referred to the Turtle Rescue Team (TRT).

**Timeline**	**Day 1**	**Day 5**	**Day 12**	**Day 13**	**Day 14**	**Day 16**	**Day 39**	**Day 40**
Treatment and diagnostics	Ketoprofen [2 mg/kg q24hrs IM 3 days]. Ceftazidime [20 mg/kg q96hrs IM 4 doses].	FNA and skin biopsy performed under sedation.	Cytology and histopathology confirm pigmented fungal infection. Ceftazidime 20 mg/kg q96hrs IM 4 doses.	Fluconazole 21 mg/kg IV (initial dose).	Fluconazole mg/kg PO SID 30 days.	Medication was injected into a mealworm to feed the patient.	PCV 17% TS 8.0 g/dL Lesions from OS and right foot did not improve. CT scan confirmed spread to the lungs and coelom cavity.	Euthanasia.

Post-mortem evaluation revealed the adipose tissue stores (visceral and subcutaneous) were markedly reduced with mild to moderate, diffuse skeletal muscle atrophy. There was a moderate expansion of the left periorbital tissue by an approximately 2.0 × 2.0 × 0.7 cm, soft, tan to gray, multilobulated mass which obscured the left orbit and partially covered the globe (Figure 1). Similar spherical ovoid masses were found located at the ventral aspect of the right forefoot (1.5 × 1.5 × 0.8 cm), ventricular epicardium (0.4 cm in diameter), both lung lobes (up to 0.3 in diameter) and scattered throughout the coelomic cavity, specifically in regions of lymphatic trunks and sinuses (2.0 × 1.0 × 1.0 cm). On cut surface, these masses had fine, brown-black stippling.

**Figure 2:**
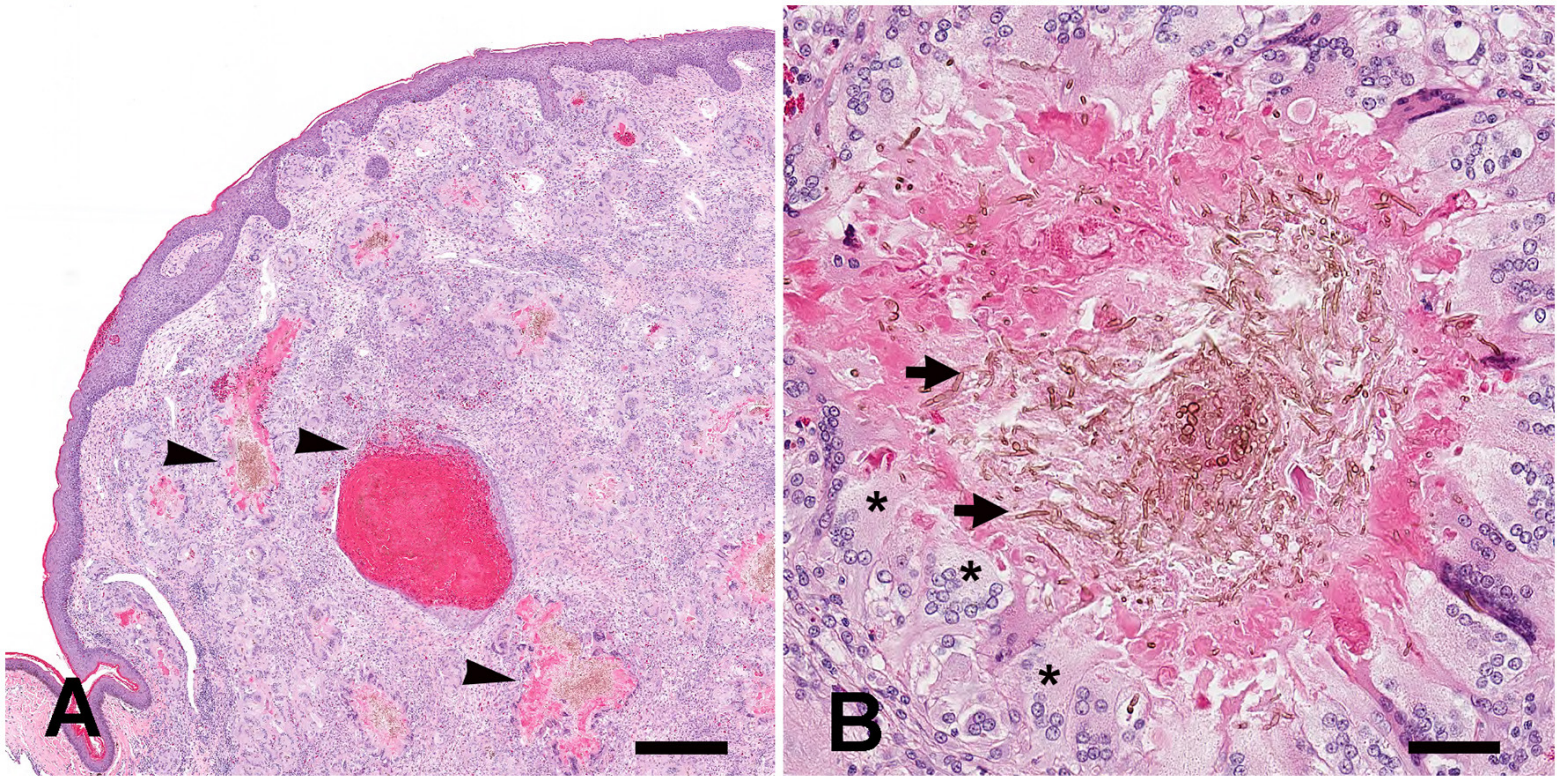
Systemic *Exophiala equina* infection in an Eastern box turtle (*Terrapene carolina carolina*). **(A)** Histologically, the dermis and subcutis of the right front foot were markedly expanded and replaced by coalescing granulomas (*arrowheads*). H&E stain. Bar size= 400 μm. **(B)** Granulomas contained large numbers of central pigmented fungal hyphae and yeast-like cells mixed with eosinophilic necrotic debris (*arrows*). Hyphae measured approximately 5 um to 7 um in diameter with parallel walls and regular septation. Yeast-like cells were irregularly ovoid and ranged from 7 um to 20 um in diameter and occasionally formed chains (pseudohyphae). Fungal organisms were surrounded by large numbers of Langhans multinucleated giant cells (*asterisks*), epithelioid macrophages. H&E stain. Bar size= 50 μm.

Histologically, the dermis, subcutis of the right front foot, and left periocular subcutaneous tissue were markedly expanded and replaced by coalescing granulomas (Figure 2A) containing abundant central pigmented fungal hyphae and yeast-like cells, single or forming chains with vesicle swellings mixed with eosinophilic necrotic debris (Figure 2B). Hyphae measured ~5–7 μm in diameter with parallel walls and regular septation. Yeast-like cells were irregularly ovoid and ranged from 7 μm to 20 μm in diameter and occasionally formed chains (pseudohyphae). Fungal organisms were surrounded by large numbers of Langhans multinucleated giant cells, epithelioid macrophages, and fewer heterophils which were further surrounded by large numbers of lymphocytes and plasma cells. In the overlying epidermis, the foot mass is focally ulcerated and replaced by abundant necrotic debris, hemorrhage, heterophils, fungal hyphae and yeast, and myriad coccobacilli. Moderate to marked hyperkeratosis was observed on the overlying lesser affected epidermis. Similar histological findings were found upon histological examination of the masses located at the ventricular epicardium, lungs, and coelomic cavity.

A swab of the periocular mass was submitted for fungal culture and the isolate was sent to the Fungal Testing Laboratory, University of Texas Health at San Antonio, Texas (FTL) for identification and antifungal sensitivity testing (UTHSCSA DI23-28). The isolate was presumptively identified as an *Exophiala* species based on phenotypic characteristics i.e., restricted suede black colonies on potato flakes agar (PFA) and yeast-like cells shown from slide culture mounts and direct tease mounts for microscopy. A subculture was submitted for sequencing of the internal transcribed spacer region (ITS) and the D1/D2 domain of the large subunit of the nuclear rDNA (LSU). The sequences were used to perform BLASTn searches to compare with available sequences of *Exophiala* species in GenBank (https://blast.ncbi.nlm.nih.gov/Blast.cgi). The ITS2 portion of the sequence was also subjected to a barcode BLAST using an updated Barcode identifier database for herpotrichiellaceous black yeasts and relatives (37, 38).

Top matches in BLASTn searches (accessed December 18, 2020) for ITS were *Exophiala equina* CBS 119.13^T^ (100%), *Exophiala tremulae* CBS 129355^T^ (99.03%), *Exophiala radicis* CBS 140402^T^ (98.63%) and top matches for LSU were *Exophiala equina* CBS 128222 (99.84%), *Exophiala equina* CBS 115143 (99.84%), *Exophiala pisciphila* CBS 100.68 (99.84%). Barcode analysis using the updated Barcode identifier database for herpotrichiellaceous black yeasts and relatives resulted in a 100% match with *Exophiala equina* CBS 119.13^T^ confirming the identity of UTHSCSA DI23-28 as *Exophiala equina*.

## 3. Discussion and literature review

Phaeohyphomycosis is a subcutaneous or systemic infection caused by melanized fungi characterized by the presence of yeast cells, septate hyphae, or pseudohyphae morphologies within the necrotic tissue sections (39). Fungi genera incriminated as causative agents of phaeohyphomycosis include *Exophiala, Hormonema, Dreschlera, Curvularia, Chaetomium, Phoma, Alternaria, Cladosporium, Madurella, Fonsecaea, Cladiophialophora*, and *Bipolaris* (40, 41).

Systemic infections can be categorized as disseminated if multiple organs are affected or localized when targeting a single organ. The two more commonly affected organs are the brain and lungs (2).

*Exophiala equina* belongs to the *salmonis*-clade with other related species including *E. psychrophila, E. opportunistica, E. salmonis, E. pisciphila, E. aquamarina, E. cancerae*, and *E. brunnea* (42). *E. equina* is isolated from water sources and watery environments, including drinking water, a cooling system on a packaging machine, the tubing of a gelly installation, silica gel, and washing of *Tilia* roots, among others (1). *E. equina* has been isolated from human patients from the Netherlands, Germany, Denmark, and the United States (42).

The diagnosis of *Exophiala equina* infection and black yeasts mycosis in general is a combined approach between culture, direct microscopy, histopathologic examination of clinical samples, and molecular analysis. Melanin is consistently present among *Exophiala* species, in the vegetative and reproductive stages, thus colonies are typically olivaceous, with dark gray or black shades on culture. This color is attributed to the presence of dihydroxynaphthalene (DHN)-derived melanin (1, 43). The growth of these fungi is invariably slow. *Exophiala* is the only genus that can produce a yeast-like phase among the *Chaetothyriales* (1, 43). Microcyclic conidiation produces the yeast-like appearance of *Exophiala* species. The liberated cells inflate and reproduce by budding, and change to hyphae by binding inflated cells, this is known as “tortulose mycelium” (1). *Exophiala* species have subtle distinctive morphological features; e.g., *E. spinifera* has long conidiophores with long annellated zones, *E. dermatitidis* has very short annelated zones next to each other in a single cell and *Exophiala equina* has ellipsoidal conidia and thick-walled, non-deciduous chlamydospores (44). DNA sequencing is required for accurate identification. BLASTn searches in GenBank are also not reliable for accurate identification because many sequences in the GenBank may represent strains that are incorrectly identified. A recently discovered fragment of about 50 bp in the ITS2 region that can be used for barcode analysis to differentiate *Exophiala* and other herpotrichiellaceous species and allows for accurate identification to the species level (37). Since the strains list for the barcode database was published in 2013, the database needs to be updated every time it is used so newly described *Exophiala* and other herpotrichiellaceaous species are included.

Phaeohyphomycosis requires a long, multimodal therapy combining antifungal drugs, surgery, thermotherapy, and chemotherapy in humans (2). Phaeohyphomycosis has been treated historically with a combination of antifungal agents, however, there is no standard therapy. Recently itraconazole, voriconazole, and posaconazole demonstrated consistent *in vitro* activity (2). Several protocols are mentioned in human literature, including a triple combination of amphotericin B, flucytosine, and itraconazole or the recent use of echinocandins (caspofungin, micafungin, and anidulafungin) (2). Itraconazole alone or in combination with Terbinafine is a commonly used protocol in human patients to treat black yeasts (2).

In general, phaeohyphomycosis is seldom reported in reptiles; however, there is a strong representation of box turtles among these cases, suggesting that this species is particularly susceptible or is just a coincidence due to the popularity of this animals as pets (40). *Exophiala* reported infections in dogs, fish, and cats are usually fatal or require euthanasia (4).

The knowledge of antifungal therapy for phaeohyphomycosis in reptiles is limited. Among the several drug families of antifungal agents, only the kinetics of azole drugs are described in reptiles (45). The most frequently used antifungals in reptiles are polyene macrolides (amphotericin B and nystatin) and azoles (ketoconazole, itraconazole, thiabendazole, fluconazole) (45). Fluconazole is the treatment of choice in reptiles, due to its effectiveness against numerous fungal species (4). The pharmacokinetic properties of fluconazole were investigated in six juvenile loggerhead sea turtles (*Caretta caretta)* (35). In this study, the authors concluded that fluconazole can be used at a dose of 10 mg/kg every 5 days after a loading dose of 21 mg/kg maintaining a desired stable concentration to effectively treat fungal infections (35). Fluconazole was used as a treatment for carpal osteomyelitis in a Kemp's ridley sea turtle (*Lepidochelys kempii)* caused by an unidentified non pigmented septate fungal hyphae in combination with *Nocardia* sp. (34). There was resolution of the lesions after 1 year of therapy, using Fluconazole 2.5 mg/kg SC q24hr and azithromycin (34). Fluconazole was used in this case, and a poor response of the lesions was noted, like the previous case report in a Galapagos tortoise (4). In that case, the treatment was for 3 days after enucleation of the left eye at a dose of 2 mg/kg PO SID (4). The necropsy confirmed a systemic phaeohyphomycosis with granulomatous inflammation within the lungs, epicardium, myocardium coelom, liver, kidney, spleen, oral cavity skeletal muscle, and thyroid (4). In the case reported herein, a poor response to the treatment was documented after 27 days of fluconazole PO, 5 mg/kg, q24 hrs after an initial loading dose of 21 mg/kg IV. Other reported treatments in turtles include combination therapy of oral terbinafine hydrochloride and a topical suspension of fluconazole in combination with surgical debridement (6). The treatment was for 17 months after the clinical presentation and was discontinued due to the patient's stable clinical condition and response to the treatment, however in this case the infection was caused by *E. oligospermia* and did not extend to the internal organs (6). Treatment was not pursued in the reported case of *Exophiala jeanselmei* infection in an Eastern box turtle (*Terrapene carolina carolina*) (5). In our case, we speculated that the disseminated disease precluded the clinical outcome of the patient and the fluconazole efficacy. Fluconazole plasma concentration above 8 μg/ml is targeted on therapies for fungal infections (34, 35), however, no measurements were performed in this case. There is no information available on the treatment of *Exophiala equina* infection in a horse.

There are several limitations in this case. For instance, this was a free-ranging animal, thus critical information regarding husbandry, diet, immune status, or previous disease was unavailable, unlike pet animals. The incidence of infections caused by *Exophiala equina* is so low that precludes the identification of specific risk factors in turtles. Although the fungus is isolated from water sources (42); the reported cases start with a cutaneous and subcutaneous infection that progresses to systemic fungi infection. The explanation of how the fungi progress from cutaneous infection to systemic remains unclear. Pathogenic melanized fungi have several virulence factors that enhance virulence and pathogenicity and can contribute to this phenomenon. The most important factors include melanin, cell polymorphism, cell adhesion, and hydrophobicity (43). Thermotolerance is an important virulence factor that allows melanized fungi to infect different hosts. Differences in maximum growth temperatures in *Exophiala* species establish the predilection for cold-blooded and warm-blooded hosts (43). *Exophiala equina* growth at a minimum of 4^o^C, optimum growth at 24–30^o^C, maximum of 33–36^o^C, and no growth at 37^o^C (42). These important features may explain why *E. equina* infections in warm-blooded hosts take place in the extremities. In humans, even though *E. equina* does not grow at 37^o^C, it can cause superficial cutaneous and subcutaneous infections in humans, but not disseminated infections into visceral organs. The only description of *Exophiala equina* in a domestic animal was causing a localized infection in a distal limb of a horse, like humans (3). The two cases reported in turtles (including the reported case herein), presented disseminated disease with hematogenous spread affecting visceral organs, and the left orbit. The affected animals have a chronic course of the disease, and the underlying initial insult cannot be determined. Traumatic inoculation is a common route of infection for many black yeasts (44). We presumed traumatic inoculation of the agent took place in this case. In cold-blooded vertebrates, predisposing factors include transportation adjacent to basins, stress under aquarium conditions, environmental changes, and pollution (2).

## 4. Conclusion

*Exophiala equina* is a melanized fungus that causes phaeohyphomycosis in animals and humans. This agent was described for the first time a century ago and is infrequently reported in the veterinary literature, and consists solely of three case reports including the reported case herein. In turtles, the clinical presentation manifests as a systemic infection with hematogenous spread and a mortality rate of 100%. Treatment with fluconazole has been unsuccessful. Although it is zoonotic, this agent does not grow at 37^o^C, and this is reflected in the clinical presentation in humans and mammals (skin and subcutaneous infection). The limitations of this case are related to the scant available literature, the undetermined route of infection, and the animal's background. This report bolsters the literature on *Exophiala* in reptiles and helps bring awareness of this pathogen and the clinical signs it produced to veterinary clinicians working with turtles and other reptiles. Unfortunately, the treatment was not successful in controlling the infection, and euthanasia was elected. Patient size and finances further limited reasonable efforts. The strengths of this case include the detailed clinical and pathological findings associated with the disease, the use of therapeutic supported by the literature, and the comprehensive multimodal approach which includes a combination of culture, histopathology, and molecular diagnostics to aim for a definitive diagnosis. Molecular diagnostic methods such as the sequencing of the ITS and proteomics such as MALDI-TOF MS, are necessary to correctly identify *Exophiala* species. The major takeaway lesson from this case is that if a diagnosis of *Exphiala* is confirmed the prognosis should be guarded and euthanasia might be the most reasonable clinical course. For those clinicians electing chemotherapy drugs other than fluconazole should be selected. Further experimental studies are required to have a more comprehensive understanding of its pathogenesis in animals and improve the therapeutic approach.

## Data availability statement

The original contributions presented in the study are included in the article material, further inquiries can be directed to the corresponding author.

## Ethics statement

Ethical review and approval were not required for this case, because the turtle was submitted for routine diagnostic postmortem examination to the Department of Pathobiology and as such is not subject to animal ethics guidelines.

## Author contributions

CC and GL followed the clinical case. TN performed the postmortem examination, sample collection, final post-mortem report, contributed to the design, supervised the study, and critically revised and edited the manuscript. NW and CC-G performed taxonomic and molecular identification of the agent. DB and SA prepared the manuscript and literature review. SA, DB, CC, GL, NW, CC-G, and TN reviewed the final submission. All authors read and approved the final manuscript.
